# Quantitative HBsAg as a biomarker for disease staging in untreated chronic hepatitis B patients: a cross-sectional study

**DOI:** 10.3389/fmed.2026.1857118

**Published:** 2026-08-10

**Authors:** Soumaya Mrabet, Imene Handous, Oussama Trabelsi, Yassmine Maatouk, Raida Harbi, Imen Akkari, Neila Hannachi, Elhem Ben Jazia

**Affiliations:** 1 Gastroenterology Department, University Hospital of Farhat Hached, Faculty of Medicine of Sousse, University of Sousse, Sousse, Tunisia; 2 Laboratory of Microbiology, University Hospital of Farhat Hached, Faculty of Medicine of Sousse, University of Sousse, Sousse, Tunisia

**Keywords:** hepatitis B, HBsAg, viral load, fibrosis, threshold

## Abstract

**Background and aims:**

Chronic hepatitis B virus (HBV) infection is a major issue in North Africa, where HBeAg-negative profiles are predominant. Distinguishing between chronic infection and active chronic hepatitis using conventional markers is sometimes difficult. Our study assessed the correlation of HBsAg with viral load and liver fibrosis, as well as its performance in distinguishing the different evolving phases of chronic HBV infection.

**Approach and results:**

We conducted an analytical cross-sectional study that included 90 HBeAg-negative treatment-naïve patients. All patients underwent biological assessment, HBsAg quantification, viral load, and FibroScan®. Among the 90 patients (mean age: 47.5 ± 11 years; 52.6% women), 65.6% were in the low-replicative chronic infection phase. The median viral load was 328.5 IU/mL, and the median HBsAg level was 213.5 IU/mL. A strong positive correlation was observed between HBsAg and HBV DNA levels (*r* = 0.721, *p* < 0.001). HBsAg levels showed a weak but significant association with liver fibrosis (*p* = 0.007, AUC = 0.631). The performance of HBsAg in differentiating low versus high replicative phases was excellent (AUC = 0.819; *p* < 0.001), with an optimal threshold of 450 IU/mL (sensitivity: 81.3%; specificity: 77.6%). In multivariate analysis, HBV DNA and AST were independently associated with disease phase, whereas qHBsAg was not an independent predictor.

**Conclusion:**

Quantitative HBsAg measurement is a relevant tool for refining the stratification of untreated HBeAg-negative patients as a complementary surrogate marker. When combined with viral load, transaminases, and FibroScan®, HBsAg could contribute to optimized monitoring and better resource allocation.

## Introduction

Chronic Hepatitis B Virus (HBV) infection remains a global health priority, affecting over 250 million individuals and serving as a primary driver of cirrhosis and hepatocellular carcinoma (1). Chronic HBV infection is a dynamic process resulting from the interaction between viral replication and host immune responses. According to the European Association for the Study of the Liver (EASL), chronic HBV infection is classified into distinct phases based on hepatitis B e antigen (HBeAg) status, serum HBV DNA levels, alanine aminotransferase (ALT) activity, and the severity of liver disease. These phases include HBeAg-positive chronic infection, HBeAg-positive chronic hepatitis, HBeAg-negative chronic infection (CI), and HBeAg-negative chronic hepatitis (CH) (2).

Among these phases, distinguishing HBeAg-negative CI from HBeAg-negative CH remains a critical clinical challenge because only patients with active hepatitis generally require antiviral therapy to prevent disease progression. However, this distinction is often complicated by the fluctuating nature of conventional biomarkers. Both serum HBV DNA levels and ALT activity may vary substantially over time, frequently necessitating prolonged follow-up before a definitive classification can be established (2).

Hepatitis B surface antigen (HBsAg) is the hallmark serological marker of HBV infection and is produced from both transcriptionally active intrahepatic covalently closed circular DNA (cccDNA) and HBV DNA sequences integrated into the host genome (3). Quantification of circulating HBsAg (qHBsAg) therefore provides information that complements serum HBV DNA measurements, reflecting not only viral replication but also the overall transcriptional activity of infected hepatocytes. Consequently, qHBsAg has emerged as a promising biomarker for disease stratification in chronic HBV infection (4). Current guidelines acknowledge its potential utility, particularly in HBeAg-negative patients. A combination of HBV DNA < 2,000 IU/mL and qHBsAg <1,000 IU/mL has been proposed to identify patients with inactive infection. With reported diagnostic accuracies of approximately 85–94% in Asian and European cohorts (2). However, the performance of these thresholds may vary across populations, as the relationship between qHBsAg levels and viral replication appears to be influenced by HBV genotype (5).

Furthermore, the relationship between qHBsAg titers and hepatic fibrosis, especially when assessed using non-invasive tools such as transient elastography, remains insufficiently investigated (6).

Data from North African populations are scarce, limiting the local validation of qHBsAg-based diagnostic strategies. Therefore, this study aimed to evaluate the correlations between qHBsAg, HBV DNA, and liver stiffness measurements (LSM), and to assess the diagnostic performance of qHBsAg in distinguishing HBeAg-negative CI from HBeAg-negative CH in a cohort of treatment-naïve patients.

## Materials and methods

This cross-sectional study included treatment-naïve patients with chronic HBV infection. Patients were eligible if they were older than 18 years, had documented HBsAg positivity for more than 6 months, were HBeAg-negative and anti-HBe-positive, and had no prior exposure to nucleos(t)ide analogues (NAs) or interferon therapy. Patients with co-infection with hepatitis C virus (HCV), hepatitis D virus (HDV), or human immunodeficiency virus (HIV) were excluded. Other exclusion criteria included the presence of chronic liver diseases other than HBV infection, such as autoimmune hepatitis, primary biliary cholangitis, alcoholic liver disease, Budd–Chiari syndrome, or portal vein thrombosis, as well as hepatocellular carcinoma or decompensated cirrhosis (history of ascites, variceal bleeding, or hepatic encephalopathy).

Serum samples were collected from all participants at the time of clinical evaluation. Quantitative HBsAg (qHBsAg) was measured using an electrochemiluminescence immunoassay (ECLIA) on the Cobas e601 platform (Roche Diagnostics). HBV DNA levels were determined by real-time polymerase chain reaction using the Artus® HBV RG amplification kit (Qiagen®, Hilden, Germany), with a lower limit of detection of 3.8 IU/mL. For statistical analyses, qHBsAg and HBV DNA values were log-transformed (log10 IU/mL). A complete blood count and routine biochemical profile, including ALT, AST, ALP, GGT, bilirubin, albumin, and INR, were performed for all participants using standard automated and enzymatic methods. In addition, abdominal ultrasonography was performed in all patients to assess liver morphology and identify potential complications or hepatic steatosis.

Liver stiffness measurement (LSM) and controlled attenuation parameter (CAP) values were obtained using transient elastography (FibroScan®, Echosens). Examinations were performed by experienced operators after a minimum fasting period of 3 h. Reliability criteria included at least 10 valid measurements and an interquartile range-to-median ratio (IQR/M) ≤ 30%. The XL probe was used in overweight or obese patients according to the manufacturer’s recommendations. Examinations performed in the presence of conditions known to affect liver stiffness measurements, including marked ALT elevation, ascites, extrahepatic cholestasis, or hepatic congestion, were excluded. Liver fibrosis was staged using established LSM thresholds, with significant fibrosis (≥F2) defined as LSM ≥ 7.2 kPa, advanced fibrosis (≥F3) as LSM > 9.4 kPa, and cirrhosis (F4) as LSM > 12.4 kPa (7).

Patients were classified into two disease phases according to EASL guidelines (3). HBeAg-negative chronic infection (CI) was defined by HBV DNA levels < 2,000 IU/mL and persistently normal ALT levels (<40 U/L), whereas HBeAg-negative chronic hepatitis (CH) was defined by HBV DNA levels ≥ 2,000 IU/mL and/or elevated ALT levels. To ensure accurate phase classification and minimize the risk of misclassifying transient fluctuations, all patients had documented follow-up of at least 6 months before inclusion. Disease phase classification was based on at least two available measurements of ALT and HBV DNA levels in accordance with EASL recommendations. For patients requiring antiviral treatment, baseline classification was established using clinical, biochemical, and virological data obtained before treatment initiation.

### Statistical analysis

Statistical analyses were performed using IBM SPSS Statistics version 27.0.

Correlations between qHBsAg, HBV DNA, and liver stiffness measurements were assessed using Spearman’s rank correlation coefficient. Receiver operating characteristic (ROC) curve analysis was performed to evaluate the diagnostic performance of qHBsAg, and the area under the ROC curve (AUROC), sensitivity, specificity, predictive values, and optimal cut-off points were determined using the Youden index. Variables associated with HBeAg-negative chronic infection at a significance level of *p* < 0.20 in univariate analyses were entered into a multivariable logistic regression model to identify independent predictors. All statistical tests were two-sided, and a *p*-value < 0.05 was considered statistically significant.

### Ethical considerations

The study protocol was approved by the Medical Ethics Committee of the Faculty of Medicine of Sousse (reference number CEFMSo_0013_2025). Written informed consent was obtained from all the participants. Data confidentiality and anonymity were ensured throughout the study.

## Results

### Baseline characteristics

Ninety treatment-naïve HBeAg-negative patients were enrolled in the study. The mean age was 47.5 ± 11 years, with a slight female predominance (52.6%). The patients were classified as having HBe-negative chronic infection (CI, *n* = 59, 65.6%) or HBe-negative chronic hepatitis (CH, *n* = 31, 34.4%). Transient elastography revealed minimal fibrosis in most of the patients. Significant fibrosis was present in11.1%, advanced fibrosis in 5.6%, and compensated cirrhosis in 4.4% of patients. The baseline characteristics of the entire cohort and the comparative analysis between the subgroups are detailed in Table 1.

**Table 1:** Baseline demographic, clinical, biochemical, and virological characteristics of the study population.

Characteristic	Total population *n* = 90	HBeAg-negative chronic infection (CI) *n* = 59	HBeAg-negative chronic hepatitis (CH) *n* = 31	*p*
Age (years)	47.5 ± 11	48.66 ± 10.8	45.54 ± 11.45	0.208
Gender (M/F)	43/47	27/32	16/15	0.598
BMI (kg/m^2^)	27.83 ± 4.6	28.8 ± 4.2	25.97 ± 4.86	**0.014**
ALT (UI/L)*	20 [16–27]	18.5 [14.75–23.25]	22.5 [18–51]	**0.004**
AST (UI/L)*	22 [19–31]	22 [19–26]	30 [22.62 – 29.85]	**0.016**
ALP (UI/L)	90 ± 70.9	80.26 ± 20.66	108.7 ± 116.46	0.195
GGT (UI/L)*	20 [13–24]	19 [12.5 – 23.5]	21 [12.75–27]	0.293
Albumin (g/L)*	41 [39.5–43]	41 [40–43]	40.5 [39–42]	0.141
Total bilirubin (μmol/L)*	12 [8.25–14]	12 [9–14]	11 [7.75–17]	0.596
Platelet count (10^3^/μL)	218 ± 77	218 ± 68.7	216 ± 84	0.69
Prothrombin Time (%)*	96 [92–100]	96 [92–100]	91.2 [91–100]	0.796
HBV DNA (UI/mL)*	328.5 [0–2.740]	168 [0–558]	4.572 [2559–66.177]	**<0.001**
qHBsAg (UI/mL)*	213.5[41.96 -1477.75]	93.91 [6.1–388]	1.331 [537.9–2.877]	**<0.001**
LSM (kPa)*	4.4 [3.67 – 5.7]	4.2 [3.6-5.5]	4.7 [3.8-7.3]	**0.033**

*Data are expressed as median [interquartile range], while other variables are expressed as mean ± standard deviation. Bold values indicate statistically significant differences (*p* < 0.05).

**Figure 1:**
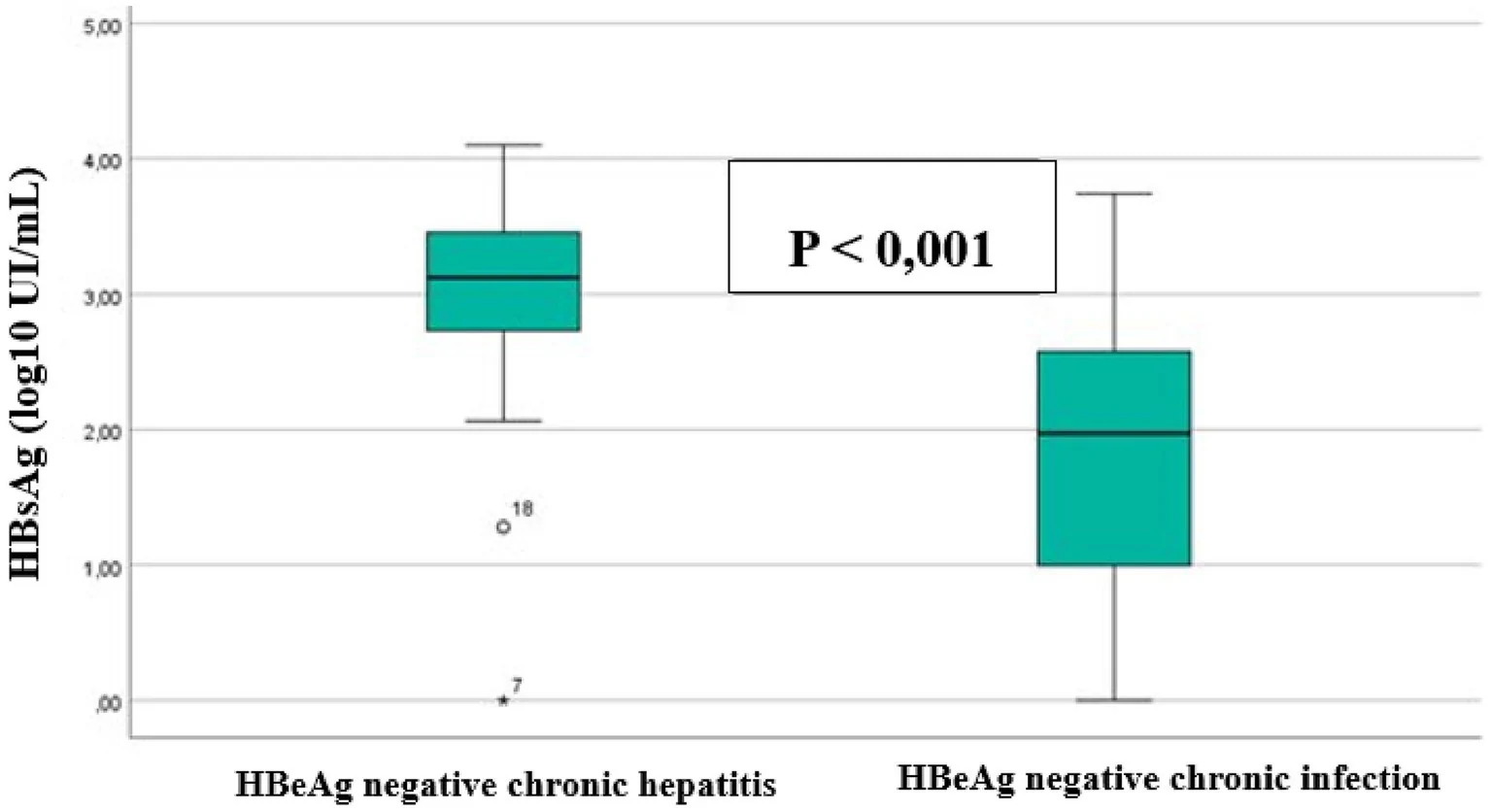
Distribution of quantitative HBsAg (qHBsAg) levels across HBeAg-negative chronic HBV infection and chronic hepatitis subgroups, showing significantly lower levels in the chronic infection phase.

Comparative subgroup analysis as shown in Table 1, showed that the CI group had a significantly higher BMI than the CH group (*p* < 0.05). Conversely, ALT, AST, HBV DNA, and LSM levels were significantly lower in the CI group (*p* < 0.05). Notably, qHBsAg levels were significantly lower in the CI group compared with those in the CH group (Figure 1).

No significant differences were observed in ALP, GGT, bilirubin, albumin, or platelet count.

### Correlation between qHBsAg and HBV DNA

**Figure 2:**
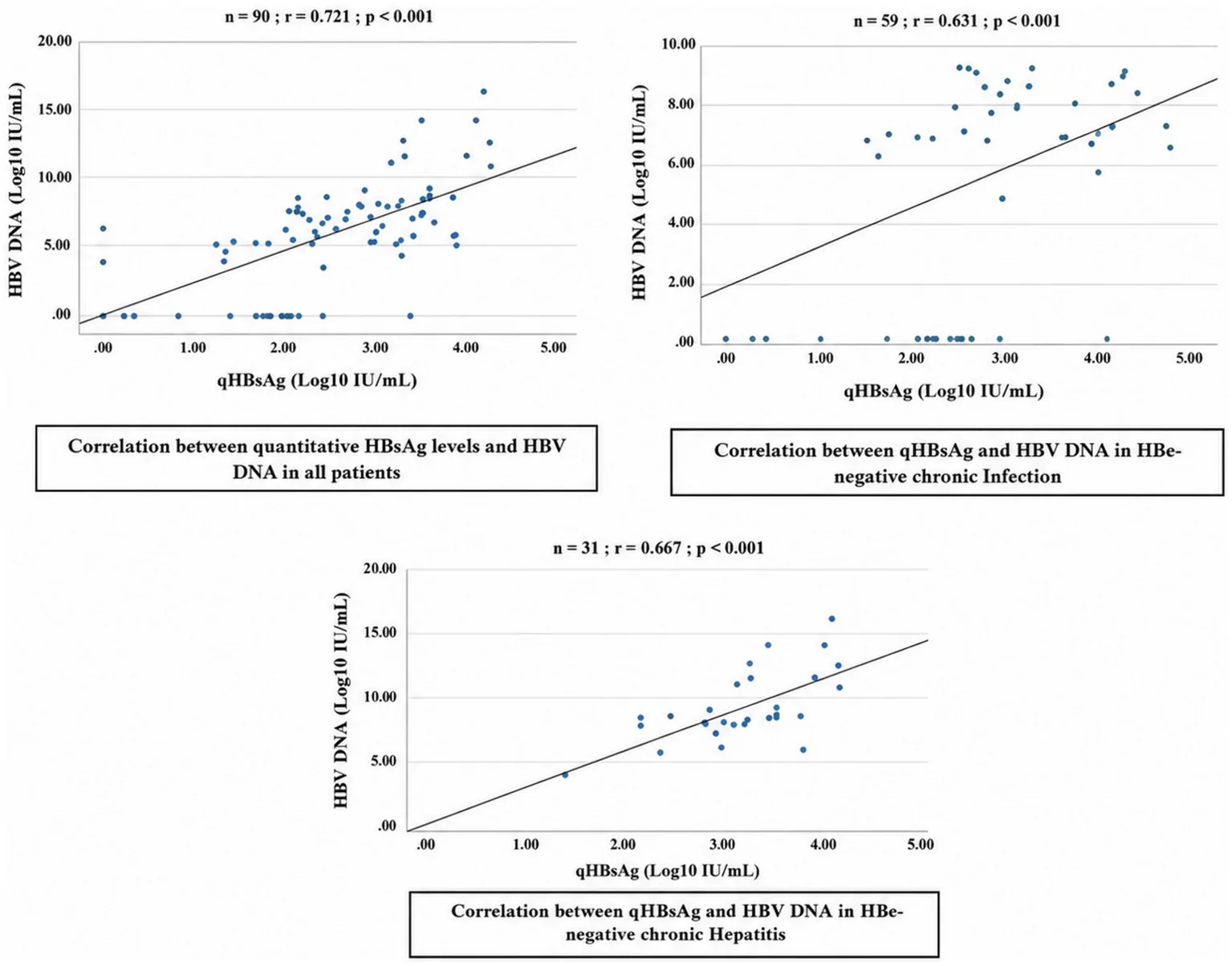
Correlation between log-transformed quantitative HBsAg (qHBsAg) and HBV DNA levels in the overall cohort and within HBeAg-negative disease phases.

A strong positive correlation was observed between qHBsAg levels and HBV DNA levels across the entire population (*r* = 0.721, *p* < 0.001). This association remained robust in both CI (*r* = 0.631, *p* < 0.001) and CH (*r* = 0.667, *p* < 0.001) subgroups (Figure 2).

### Correlation between qHBsAg and liver fibrosis

**Figure 3:**
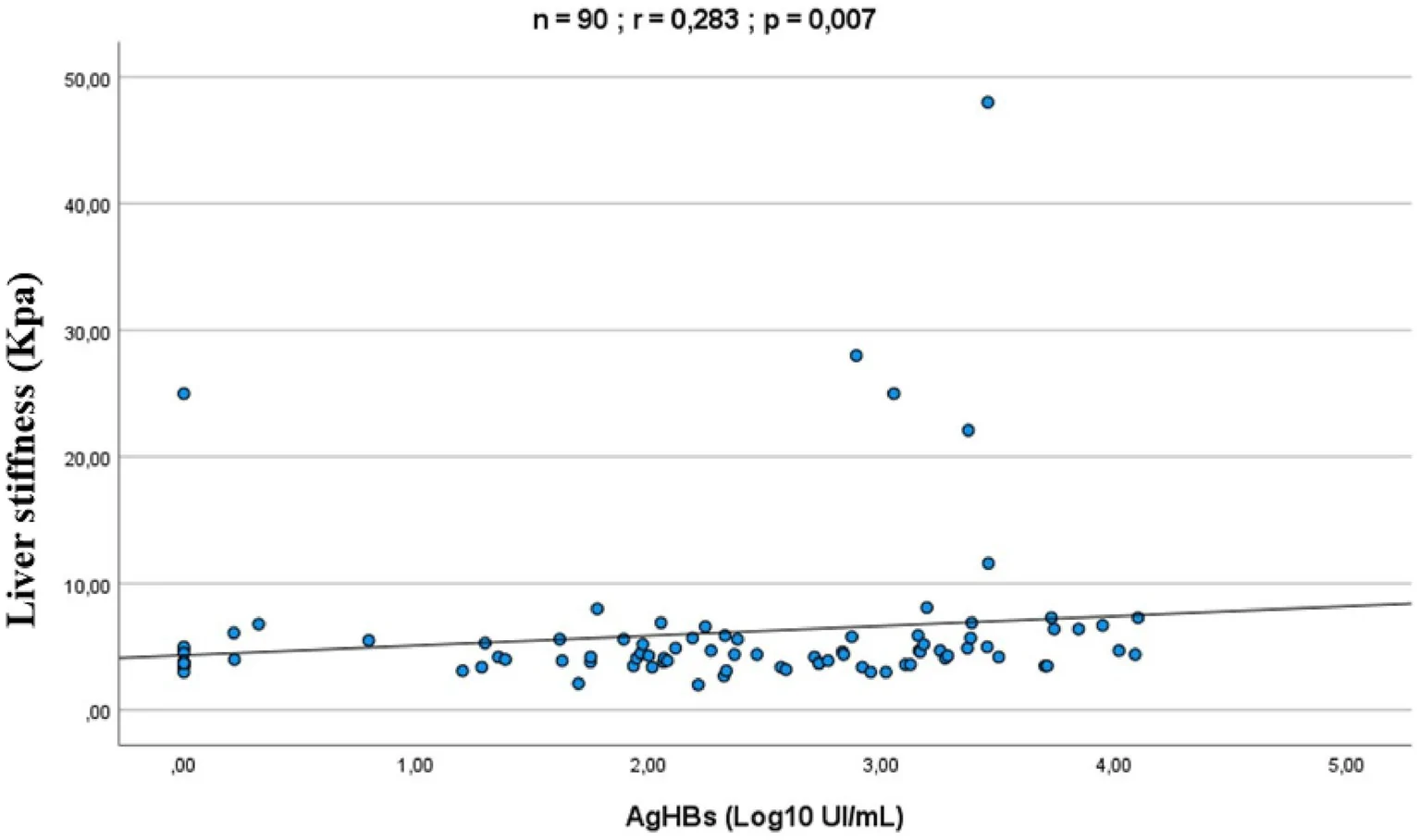
Correlation between qHBsAg levels and Liver Stiffness Measurement assessed by transient elastography, showing a weak but significant correlation.

A weak but statistically significant correlation was observed between qHBsAg and LSM (*r* = 0.283, *p* = 0.007) (Figure 3).

### Diagnostic performance of qHBsAg

**Figure 4:**
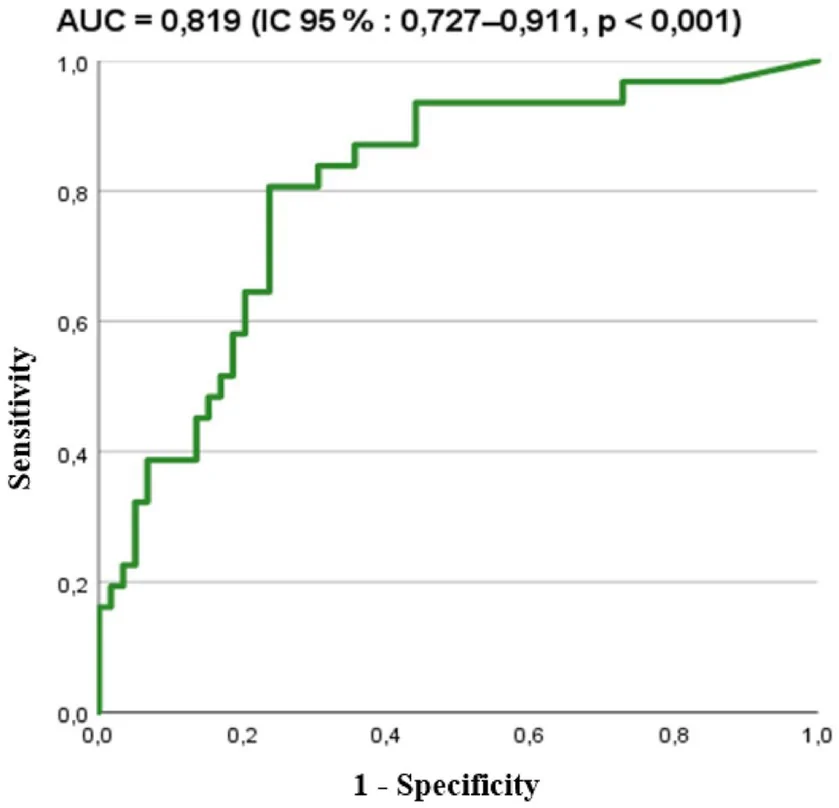
Receiver operating characteristic (ROC) curve of quantitative HBsAg (qHBsAg) for distinguishing HBeAg-negative chronic hepatitis from chronic infection, with an AUROC of 0.819.

The diagnostic utility of qHBsAg was evaluated for two clinical endpoints:

1. Phase Discrimination (CH vs CI): qHBsAg showed a high discriminative power with an AUROC of 0.819 (95% CI: 0.727–0.911; *p* < 0.001). Using the Youden index, the optimal cut-off was 450 IU/mL. At this threshold, qHBsAg yielded a sensitivity of 81.3%, specificity of 77.6%, a negative predictive value (NPV) of 88.2%, and diagnostic accuracy of 78.9% for distinguishing CH (Figure 4).
2. Fibrosis Prediction: In contrast, qHBsAg demonstrated non-significant performance in predicting significant fibrosis (AUROC 0.63, *p* = 0.174).

In multivariate logistic regression analysis (*including age, BMI, qHBsAg, HBV DNA, LSM, ALT, AST, ALP and albumin*), HBV DNA level (OR = 0.283; 95% CI: 0.147–0.544; *p* < 0.001) and AST (OR = 0.896; 95% CI: 0.839–0.957; *p* = 0.001) were independently associated with HBeAg-negative chronic infection status, whereas qHBsAg did not retain independent predictive value.

## Discussion

The clinical trajectory of HBeAg-negative chronic HBV infection in North Africa reflects a virological context that is strongly influenced by the predominance of HBV genotype D, which is the most frequently reported genotype in the region, particularly sub-genotype D7, considered a regional variant of North Africa. This sub-genotype has been consistently reported as the most frequent among genotype D strains, comprising 66% of the isolates (8, 9).

Importantly, the HBeAg-negative phenotype is mechanistically driven by the emergence of precore (PC) stop codon mutations, particularly G1896A, and basal core promoter (BCP) mutations, including A1762T/G1764A. These mutations reduce or abolish HBeAg expression while preserving viral replication, thereby constituting the molecular basis of the active HBeAg-negative chronic hepatitis phase (10, 11). Their occurrence is closely linked to HBV genotype, being particularly favored in genotype D (12).

In our study, we found that qHBsAg levels in HBeAg-negative patients were strongly correlated with HBV DNA (*r* = 0.721). This correlation is remarkably higher than the pooled coefficient of *r* = 0.29 in the largest global meta-analysis to date (13). Notably, our findings surpass the moderate correlation (*r* = 0.44) observed in previous regional reports from another center in Monastir (14).

In HBeAg-negative disease, HBsAg is synthesized from both cccDNA transcription and integrated HBV DNA (15). Our findings suggest tighter functional coupling between antigen production and replicative activity. This may reflect a lower relative contribution of replication-independent integrated DNA to the total HBsAg pool. However, since molecular analyses of mutational status were not conducted in this study, this hypothesis remains strictly speculative and requires further focused molecular investigation.

A major finding of our study was the identification of an optimized qHBsAg threshold of 450 IU/mL to discriminate HBe-negative chronic infection (CI) from chronic hepatitis (CH), achieving a clinically significant performance (AUROC = 0.819) with a high negative predictive value (88.2%). To distinguish HBeAg-negative chronic infection from HBeAg-negative chronic hepatitis, the current EASL guidelines indicate that individuals with HBeAg-negative chronic infection can be identified using a combination of HBV DNA < 2,000 IU/mL and qHBsAg <1,000 IU/mL, with reported diagnostic accuracies of 85–94% in Asian and European cohorts (16). However, our data suggest that applying this 1,000 IU/mL threshold to discriminate between HBeAg-negative chronic infection and chronic hepatitis may substantially reduce sensitivity in our population. In our series, this threshold yielded a sensitivity of only 59.4%, misclassifying nearly half of the patients with active chronic hepatitis as inactive. Although very low thresholds (<100 IU/mL) reported in recent multicenter studies may improve specificity across genotypes, our 450 IU/mL cut-off represents a pragmatic intermediate threshold balancing sensitivity and specificity (17). These findings emphasize the need for regional recalibration to optimize the sensitivity–specificity balance across diverse viral genotypes.

Nevertheless, this value must be interpreted with caution and requires external validation in larger, independent cohorts before its clinical implementation.

Regarding liver fibrosis assessment, a weak but statistically significant correlation was observed between serum qHBsAg and liver stiffness ( p=0.007 ). However, its diagnostic performance in detecting significant fibrosis was not significant (AUROC = 0.63; p>0.05 ). This dissociation indicates that in the late stages of chronic HBV infection, serum HBsAg primarily reflects ongoing viral transcriptional activity rather than cumulative immune-mediated structural damage. These findings align with those of studies in HBeAg-negative patients, where qHBsAg showed weak or absent correlations with fibrosis (18, 19).

In contrast to HBeAg-positive cohorts, −where inverse correlations between qHBsAg and fibrosis are often reported- our data confirm that qHBsAg cannot be used as a standalone surrogate for elastometric assessment (20, 21). Consequently, while qHBsAg alone has limited diagnostic utility, it may enhance multimodal algorithms combining biomarkers and elastography for non-invasive liver fibrosis evaluation.

**Figure 5:**
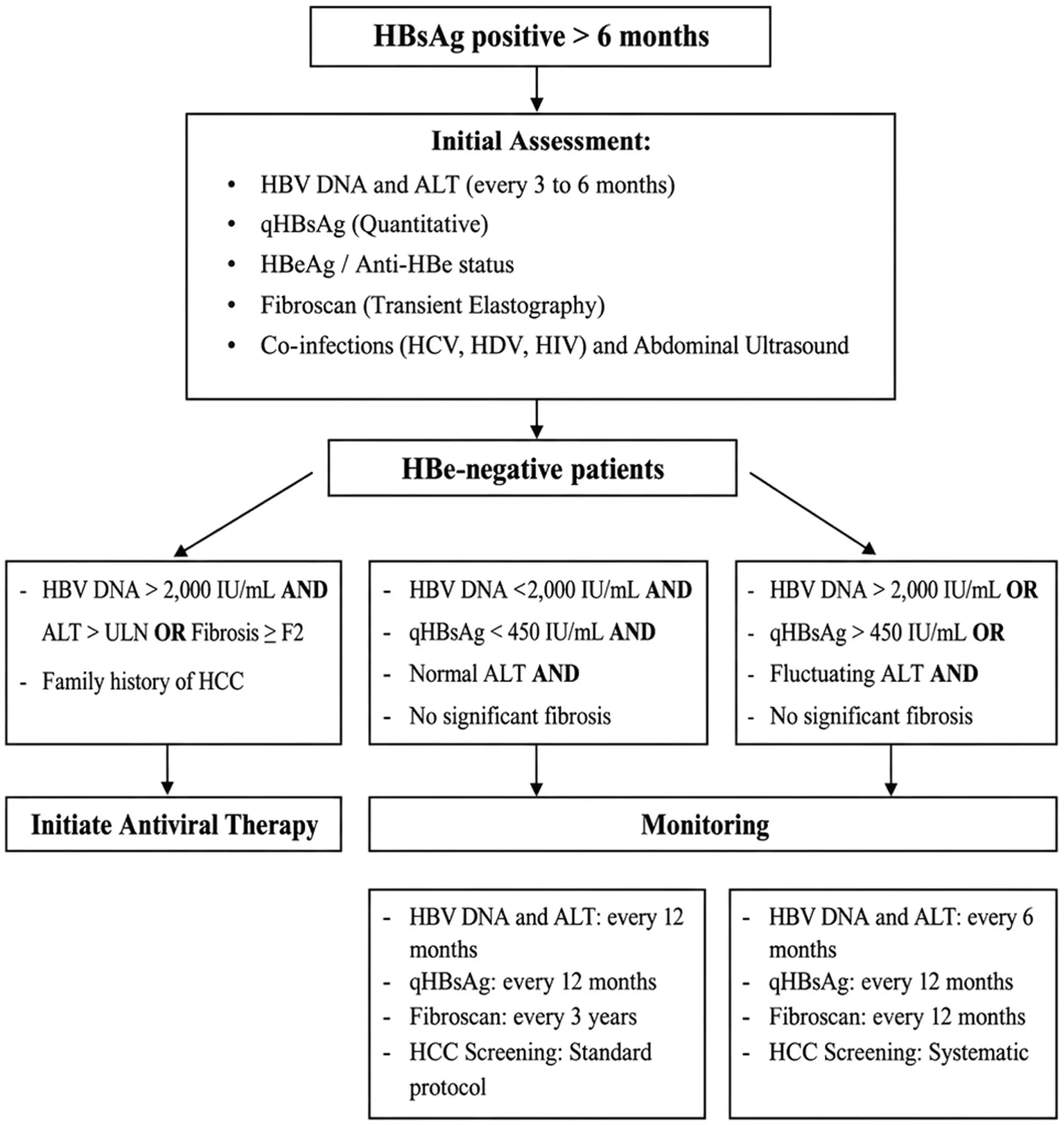
Decision-making algorithm and practical recommendations for the management of HBe Ag negative chronic hepatitis B.

Ultimately, our findings highlight an important nuance: although qHBsAg is a strong discriminative biomarker in univariate assessment, it does not emerge as an independent predictor of disease phase after adjustment for viral load and biochemical activity. This is likely related to the strong correlation observed between qHBsAg and HBV DNA in our cohort, reflecting partial collinearity between these virological markers. In this context, qHBsAg should be interpreted as a complementary biomarker with relevant diagnostic performance (AUROC = 0.819), rather than as an independent determinant of disease phase. Based on these findings, we propose an adapted management algorithm (Figure 5) inspired by the EASL 2025 guidelines. For HBeAg-negative patients:

- Chronic infection was defined as HBV DNA < 2,000 IU/mL and qHBsAg < 450 IU/mL, combined with normal ALT and absence of significant fibrosis.
- Optimized monitoring: Patients with qHBsAg > 450 IU/mL, even with low viral loads, require closer monitoring (every 3–6 months) because of the higher risk of reactivation.

### Limitations and future directions

Despite its clinical relevance, this study is limited by its monocentric, cross-sectional design, which restricts the assessment of dynamic fluctuations characterizing HBeAg-negative chronic HBV infection. Although we mitigated the risk of misclassification by utilizing at least two serial ALT and HBV DNA measurements over a minimum 6-month follow-up period for phase definition, we acknowledge that longer longitudinal monitoring, including serial qHBsAg assessments, would have provided a more precise characterization of phase transitions.

Second, the absence of liver biopsy, the histological reference standard, precludes direct correlation with tissue-based fibrosis staging. Although transient elastography is a validated non-invasive tool for fibrosis assessment, its exclusive use introduces inherent limitations, particularly its reduced accuracy for distinguishing intermediate fibrosis stages and its inability to assess necroinflammatory activity in chronic HBV infection.

In addition, the low prevalence of advanced fibrosis and cirrhosis may have introduced a spectrum effect, limiting the ability to detect stronger associations between qHBsAg levels and advanced liver disease.

Future multicenter research should evaluate whether the threshold of 450 UI/mL predicts long-term outcomes, such as HBsAg seroclearance and reduced risk of hepatocellular carcinoma, to further refine functional cure strategies in North Africa.

## Conclusion

Our findings support the clinical utility of qHBsAg as an accessible and cost-effective biomarker for managing HBeAg-negative chronic HBV infection in the North African context. While not substituting for HBV DNA quantification, qHBsAg facilitates more accurate phase discrimination through a locally optimized, albeit exploratory, threshold of 450 IU/mL. We emphasize that this cut-off is preliminary and requires external validation in larger, prospective, and multi-center cohorts before routine clinical implementation. However, as its performance in predicting fibrosis is limited, it should be integrated into a dual-track strategy, virological assessment (qHBsAg and HBV DNA), complemented by transient elastography for structural staging.

## Data Availability

The raw data supporting the conclusions of this article will be made available by the authors, without undue reservation.
